# Supplementary material for: Rapid restoration of cell phenotype and matrix forming capacity following transient nuclear softening
Source: Acta Biomater. Author manuscript; Available in PMC 2026 Apr 6. (PMC13052731; doi:10.1016/j.actbio.2025.10.007)
Supplement: supplemental materials [file NIHMS2157102-supplement-supplemental_materials.pdf]

Supplemental figures

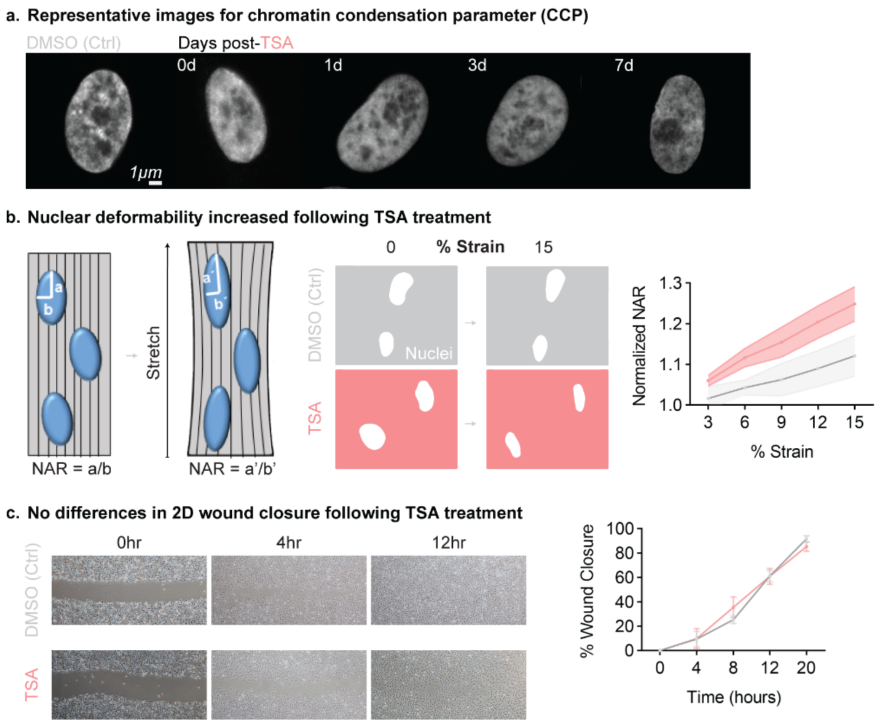

Supplemental Figure 1. (a) Representative images of high magnification DAPI-stained nuclei used for quantification of chromatin condensation parameter edge density. (b) Schematic of nuclear deformability assay and representative binarized images of DAPI-stained nuclei at 0 and 15% strain for DMSO-treated controls and TSA-treated cells. Quantification of the nuclear aspect ratio (NAR) at incremental strain steps for DMSO-treated controls and TSA-treated cells, showing increased nuclear deformability at each strain step following TSA treatment. (c) Representative images of 2-dimensional scratch assay over time for DMSO-treated controls and TSA-treated cells, and quantification of percent wound closure over time. N=5-6.

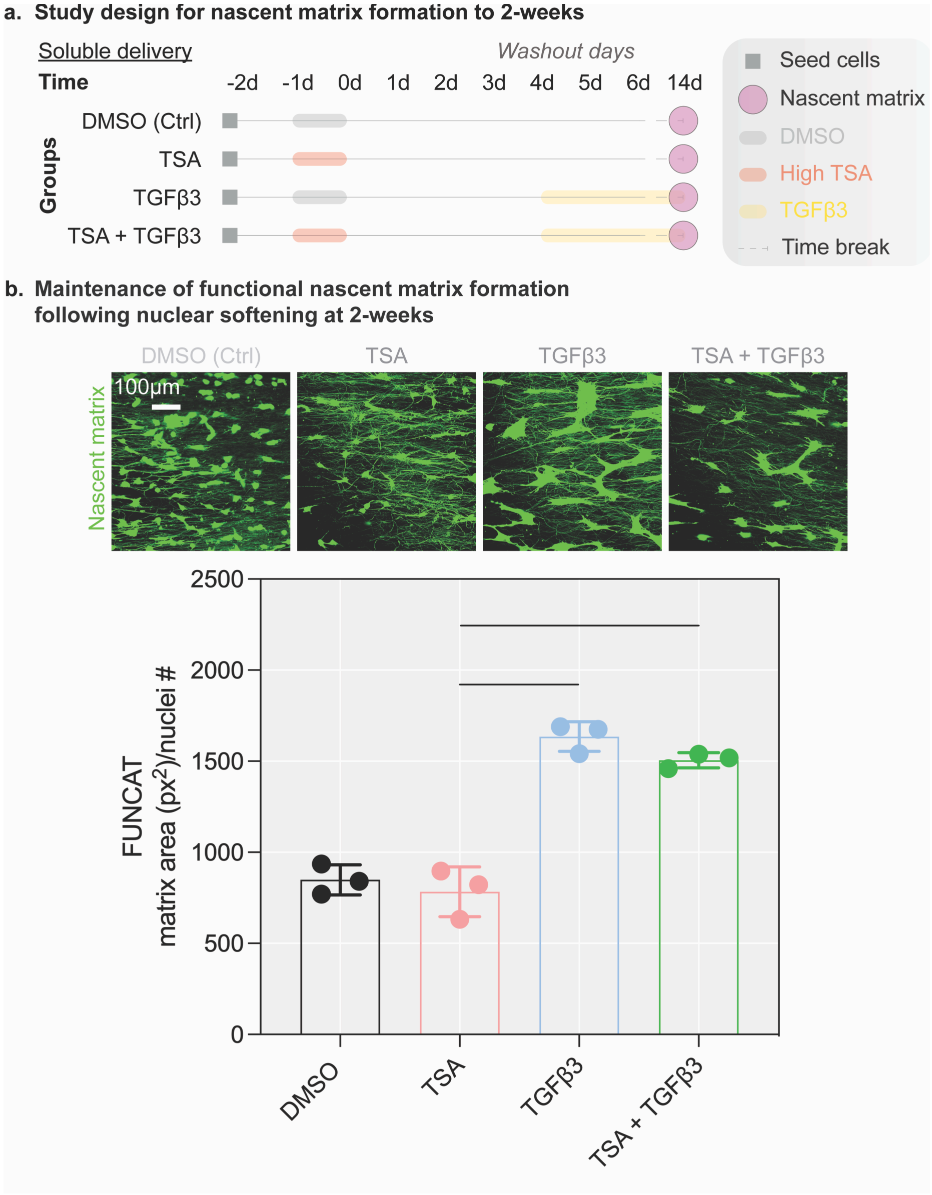

Supplemental Figure 2. (a) Study design to evaluate the functional effect of nuclear softening on nascent matrix production in porcine meniscus cells cultured on fibrous scaffolds. TSA: trichostatin A, DMSO: dimethyl sulfoxide, TGFβ3: transforming growth factor β3, Ctrl: Control. Highlighted lines: treatments of DMSO (gray), TSA (red), and TGFβ3 (yellow). Shapes: specific start and end points denoted in the legend. d: day. (b) Representative confocal images of nascent matrix at 14 days following DMSO, TSA, TGFβ3, or TSA + TGFβ3 treatment. Green: deposited nascent matrix over the culture period. Nuclei were counted via segmentation of the DAPI channel (not shown). Scale bar = 100µm. (c) Quantification of nascent matrix at 14 days following DMSO, TSA, TGFβ3, or TSA + TGFβ3 treatment. Bars: significantly different, $p<0.05$. One-way ANOVA.

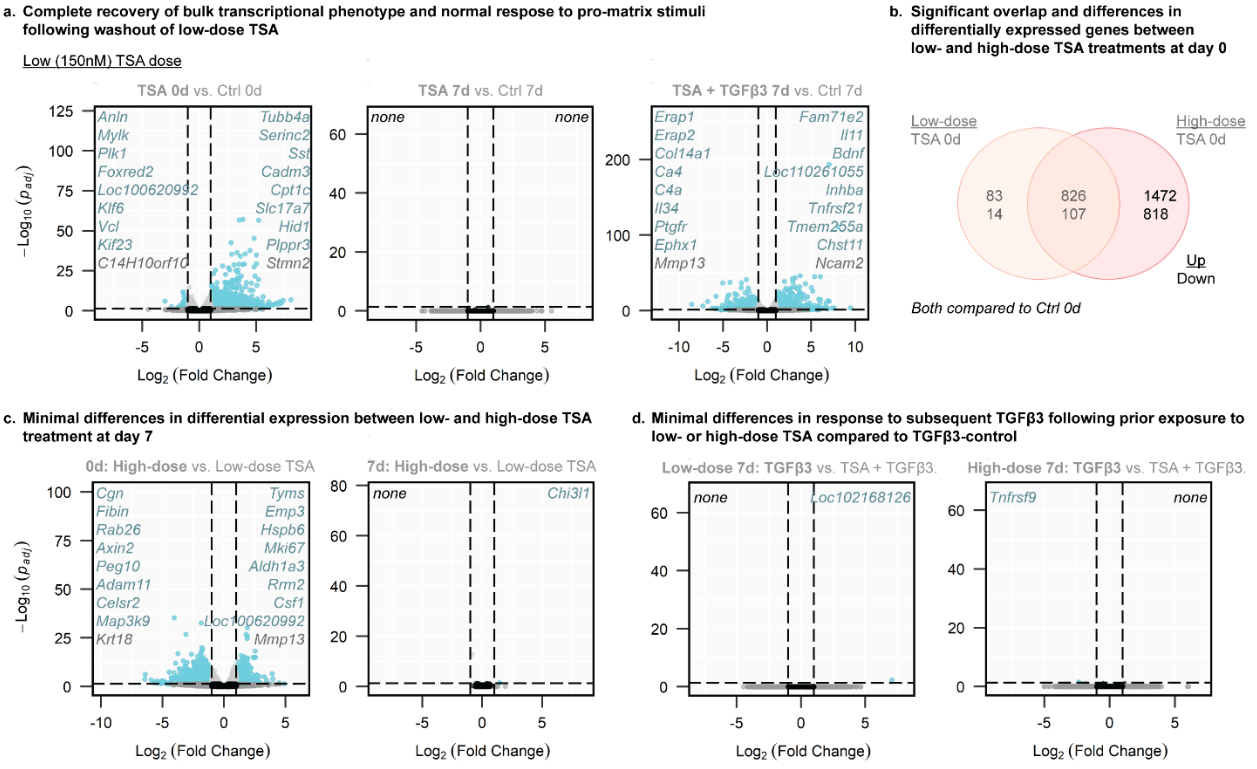

Supplemental Figure 3. (a) Volcano plots for low-dose TSA treatment groups. (b) Venn diagram comparing differentially expressed genes at day 0 following low- and high-dose TSA. Numbers indicate upregulated (top number) genes and downregulated (bottom number) genes. Non-overlapping quadrants represent unique genet sets for each group. (c) Volcano plots for high-dose compared to low-dose TSA treatment at day 0 and 7. (d) Volcano plots for TGFβ3 only compared to low- or high-dose TSA + TGFβ3 at day 7.

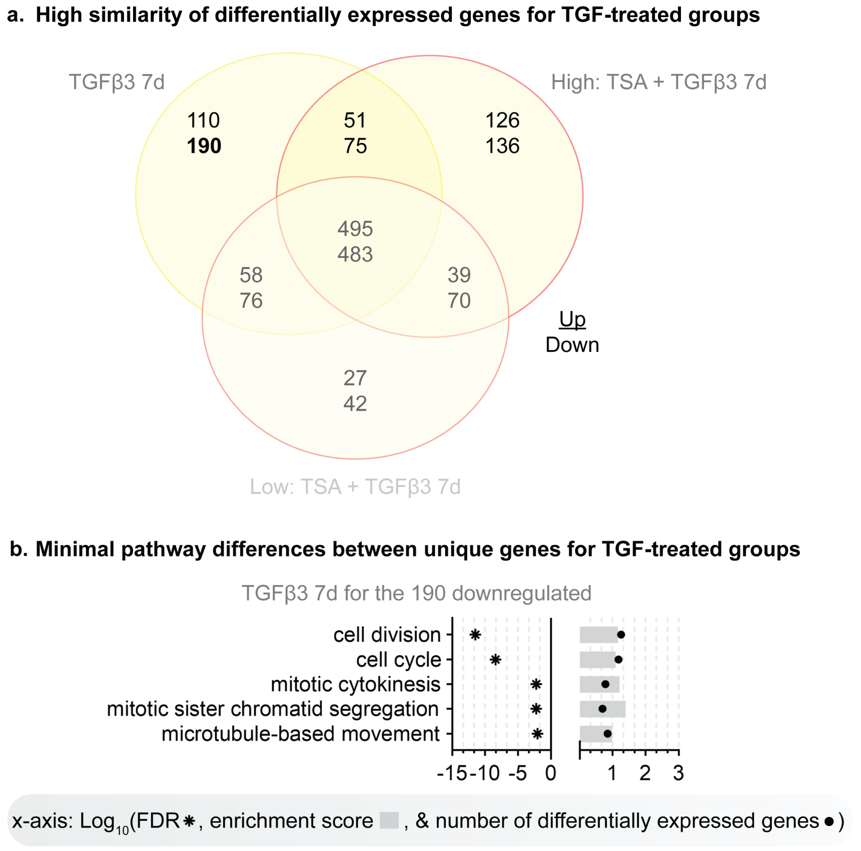

Supplemental Figure 4. (a) Venn diagram of differentially expressed genes between groups treated with TGFβ3, including TGFβ3 only, high-dose TSA + TGFβ3, and low-dose TSA + TGFβ3. Numbers indicate upregulated (top number) genes and downregulated (bottom number) genes. Non-overlapping quadrants represent unique gene sets for each group. (b) Gene ontologies for unique upregulated genes following high-dose TSA + TGFβ3. Note: The unique downregulated genes following high-dose TSA + TGFβ3 and both the up- and down-regulated genes following low-dose TSA + TGFβ3 were not significantly enriched into specific gene ontologies.

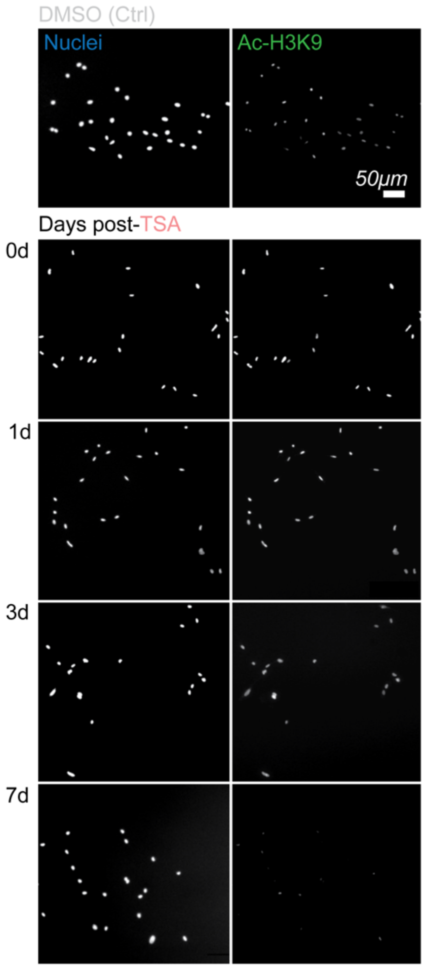

Supplemental Figure 5. Histone acetylation in DMSO-treated controls and 0, 1, 3, and 7 days post-TSA treatment. Fluorescence images showing DAPI and Ac-H3K9 and DAPI. Scale bar = 50μm.
